# New HPV Vaccines on the Market and Future Trends: A State-of-the-Art Review

**DOI:** 10.3390/vaccines14020140

**Published:** 2026-01-29

**Authors:** Utku Akgör, Bilal Esat Temiz, Murat Cengiz, Hasan Volkan Ege, Elmar Joura, Murat Gültekin

**Affiliations:** 1 Faculty of Medicine, Department of Obstetrics and Gynecology, Division of Gynecologic Oncology, Hacettepe University, 06230 Ankara, Türkiye; utkuakgor@gmail.com (U.A.); bilalesattemiz@gmail.com (B.E.T.); drmuratcengizgyn@gmail.com (M.C.); 2 Department of Gynecologic Oncology, Etlik City Hospital, 06010 Ankara, Türkiye; drvolkanege@gmail.com; 3 Department of Gynecology and Obstetrics, Comprehensive Cancer Center, Medical University of Vienna, 1090 Vienna, Austria; elmar.joura@meduniwien.ac.at

**Keywords:** HPV vaccines, next-generation HPV vaccines, immunogenicity, L2-based vaccine candidates, human papillomavirus

## Abstract

Next-generation human papillomavirus (HPV) vaccines encompass newly licensed and emerging formulations that employ alternative production platforms, expanded valency, or novel antigenic targets beyond conventional L1-based vaccines. These vaccines aim to address affordability challenges, supply limitations, and suboptimal vaccination coverage, particularly in low- and middle-income countries. This review aggregates current clinical, immunological, and programme-related evidence on newly licensed vaccines, including the World Health Organization (WHO)-prequalified bivalent formulations (Cecolin^®^ and Walrinvax^®^), the quadrivalent Cervavac^®^, and the *Escherichia coli*-derived nonavalent Cecolin 9^®^, which received national licensure in 2025. Additionally, emerging high-valency candidates in Phase I–III trials—9-valent, 11-valent, and 14-valent formulations—are critically assessed. Clinical trials demonstrate that next-generation HPV vaccines provide robust protection; for example, Cecolin^®^ showed 100% efficacy against HPV-16/18-associated high-grade squamous intraepithelial lesions (HSIL) and up to 97.8% efficacy against persistent HPV infection, while Walrinvax^®^ demonstrated 78.6% protection against CIN2+ lesions. Cervavac^®^ showed non-inferior immunogenicity compared with established vaccines. While comparative analyses of efficacy, immunogenicity, and safety indicate that these vaccines are strong alternatives to established products, robust long-term effectiveness and real-world impact data remain essential before full clinical equivalence can be definitively established. Advances in L2-based platforms further aim to broaden cross-type protection, simplify manufacturing, and enable thermostable formulations, thereby enhancing applicability in resource-limited settings. Economic evaluations demonstrating favorable cost-effectiveness emphasize the essential role of next-generation vaccines in improving access and reducing inequity. Overall, innovations in valency, technology, and delivery strategies have the potential to significantly expand global HPV prevention coverage and accelerate progress toward cervical cancer elimination.

## 1. Introduction

Cervical cancer continues to be a major health issue worldwide, especially in low- and middle-income countries (LMICs), where mortality remains high despite the World Health Organization’s (WHO’s) goals for elimination by 2030 [1,2]. Although the causal link between persistent high-risk human papillomavirus (HPV) infection and cervical cancer is well understood, the field of HPV prevention has advanced significantly since the first prophylactic vaccine was approved in 2006 [3,4].

Currently, HPV vaccines are based on virus-like particles (VLPs) composed of the major capsid protein L1, which are highly immunogenic and provide type-specific protection against the HPV genotypes included in each formulation [5]. The first-generation vaccines—bivalent (Cervarix™ (GlaxoSmithKline Biologicals, Rixensart, Belgium); 2vHPV) and quadrivalent (Gardasil™ (Merck & Co., Inc., Kenilworth, NJ, USA); 4vHPV)—revolutionised cervical cancer prevention by targeting the most oncogenic HPV types, HPV16 and 18. The second-generation nonavalent vaccine (Gardasil 9™ (Merck & Co., Inc., Kenilworth, NJ, USA); 9vHPV) expanded coverage to include five additional high-risk types (HPV31, 33, 45, 52, and 58), providing protection against approximately 90% of cervical cancer cases [6].

Several new HPV vaccines have been introduced and have received the WHO prequalification. These vaccines aim to improve access, affordability, efficacy, and immune response, particularly in low-resource settings [7]. Recently approved in China and other low- and middle-income countries, two bivalent vaccines, Cecolin™ (Xiamen Innovax Biotech Co., Ltd., Xiamen, China) and Walrinvax™ (Yuxi Zerun Biotechnology Co., Ltd., Yuxi, China), target HPV 16 and 18 [8]. In 2022, the Serum Institute of India launched Cervavac™ (Serum Institute of India Pvt. Ltd., Pune, India), India’s first domestically produced quadrivalent HPV vaccine [8]. Meanwhile, Chinese companies such as Innovax and Walvax are developing new HPV vaccines, with several candidates in advanced clinical trials [6,8]. Notably, the second-generation nonavalent vaccine, Cecolin 9™, developed using an *Escherichia coli* platform, has completed Phase II-III trials and was recently recommended by the China NMPA as an alternative to Gardasil 9™ [9].

To address existing gaps in global cervical cancer prevention, there is a growing need to better understand the rapid development of HPV vaccine technology, particularly as new products emerge with the potential to expand coverage in low- and middle-income countries. Achieving the WHO 2030 cervical cancer elimination targets will depend not only on scaling up existing vaccination programs but also on developing next-generation vaccines that can overcome persistent challenges such as accessibility, affordability, and population-level protection. This review summarizes recent developments in HPV vaccines, focusing on newly licensed and emerging formulations and assessing their immunogenicity, efficacy and cost-effectiveness.

## 2. Materials and Methods

### 2.1. Review Design

This manuscript was conducted as a narrative (state-of-the-art) review. The aim was to provide a comprehensive overview of newly licensed and emerging HPV vaccines, including prophylactic vaccines, L2 minor capsid protein vaccine platforms, therapeutic vaccine approaches, and their clinical, regulatory, and economic implications.

### 2.2. Literature Sources and Search Approach

Relevant literature published between January 2006 and December 2025 was identified through targeted searches of PubMed (National Center for Biotechnology Information, Bethesda, MD, USA), Web of Science (Clarivate Analytics, Philadelphia, PA, USA), and Scopus (Elsevier, Amsterdam, The Netherlands). Searches were performed using Medical Subject Headings (MeSH) terms and free-text keywords related to HPV vaccination, including “HPV vaccine”, “human papillomavirus vaccination”, “new HPV vaccines”, “L2-based HPV vaccines”, “therapeutic HPV vaccines”, “HPV vaccine immunogenicity”, “HPV vaccine efficacy”, and “cost-effectiveness of HPV vaccines”. Boolean operators (AND, OR) were used to refine the search strategy.

In addition to peer-reviewed publications, regulatory and policy documents from international and national authorities—including the World Health Organization (WHO, Geneva, Switzerland), European Medicines Agency (EMA, Amsterdam, The Netherlands), U.S. Food and Drug Administration (FDA, Silver Spring, MD, USA), and China National Medical Products Administration (NMPA, Beijing, China)—were reviewed. Information on ongoing and completed clinical trials was obtained from ClinicalTrials.gov (U.S. National Library of Medicine, Bethesda, MD, USA) and other publicly accessible trial registries. Literature was considered based on clinical relevance, with priority given to robust clinical trials, while very small-scale studies (*n* < 50) were not emphasized to ensure the reliability of the synthesis.

## 3. New Licenced Vaccines

HPV vaccines have been formulated to contain L1 capsid proteins that can self-assemble into VLPs. These VLPs mimic the structure of authentic virions but do not contain live biological agents or viral DNA [10]. It is clearly shown in the literature that it is highly effective in protecting against the most common oncogenic HPV types, leading to a significant decrease in infection rates and a decrease in the incidence of high-grade cervical lesions [11]. Despite advances, HPV vaccines do not protect against all oncogenic HPV types, and some associated infections remain untreatable. Furthermore, significant inequities in vaccine access, particularly among low-income populations, are a significant concern. Financial constraints, logistical challenges and inadequate health infrastructure may delay large-scale vaccination efforts and exacerbate global health inequalities [12].

The development of new HPV vaccines has begun to accelerate significantly in recent years to facilitate access, especially in Low- and Middle-Income Countries. In this context, HPV vaccines produced by biotechnology companies, especially in the China and India, which constitute the majority of the world’s population, have begun to go through the licensing process, allowing for wider distribution. In 2021, the WHO prequalified Cecolin, which targets HPV types 16 and 18, paving the way for its inclusion in immunisation programs in low- and middle-income countries (LMIC) [13,14]. Then, in 2022, the WHO prequalified Walrinvax, another bivalent vaccine targeting the same high-risk HPV types [14,15]. Similarly, in India, Cervavac, a quadrivalent vaccine targeting HPV types 6, 11, 16 and 18, received approval from the Central Drugs Standard Control Organization (CDSCO) of India and was subsequently included in the national immunization schedule of that country [16]. To provide a clear picture of the newly approved HPV vaccines worldwide, see Table 1, which shows the key features of the vaccines approved by the major regulatory agencies.

### 3.1. Cecolin (HPV-16/18 Bivalent Vaccine)

Cecolin^®^ is a bivalent HPV vaccine composed of 40 μg of HPV-16 and 20 μg of HPV-18 recombinant L1 VLPs, which are expressed in *Escherichia coli* and adsorbed onto an aluminium hydroxide adjuvant [17]. Cecolin became licensed in China in December 2019. The vaccine is administered as a two- or three-dose regimen for girls aged 9–14 years. It is administered as a three-dose regimen for persons aged 15 years and older [7].

A placebo-controlled Phase III clinical study in China involving 7372 women aged 18–45 years demonstrated that Cecolin was 100% effective in preventing HPV-16/18-associated high-grade squamous intraepithelial lesion (HSIL). Additionally, the vaccine was 97.8% effective in preventing persistent HPV-16/18 infections [18]. Another Phase III, double-blind, randomized controlled trial (NCT01735006), conducted across five clinical centres in China, enrolled 1455 women aged 18–45 years. The study confirmed the efficacy of Cecolin after 66 months of follow-up, reporting 100% vaccine efficacy (VE) against HSIL and 97.3% VE against persistent HPV-16 and HPV-18 infections [13].

The first clinical data evaluating Cecolin outside China were published in 2024, from an open-label, randomised Phase III trial comparing Cecolin with Gardasil. The study included a cohort of 1025 girls aged 9–14 years from Bangladesh and Ghana, representing populations from both Africa and South Asia. The study evaluated the immunogenicity and safety of Cecolin administered in two-dose regimens on 0- and 6-month schedules and demonstrated that it was not immunologically superior to Gardasil [19].

The performance of the vaccine over time will be monitored at 12 and 24 months. Preliminary results indicate that Cecolin has a favorable safety profile and may represent a more affordable HPV vaccine option in low- and middle-income countries, as supported by recent cost-effectiveness analyses comparing Cecolin-based programmes with Gardasil 9–based strategies in resource-limited settings. These findings led to the WHO’s decision to grant Cecolin prequalification in 2021, opening the way for its inclusion in HPV vaccination programmes worldwide [14].

### 3.2. Walrinvax (HPV-16/18, Bivalent Vaccine)

Walrinvax^®^ is a bivalent human papillomavirus (HPV) vaccine developed to protect against HPV-16 and HPV-18. It was created by Yuxi Zerun Biotechnology Co., Ltd., Yuxi, China, a subsidiary of Walvax Biotechnology Co., Ltd., Kunming, China, a China-based biotechnology company. The vaccine consists of VLPs derived from recombinant L1 capsid proteins of HPV-16 and HPV-18 produced using the *Pichia pastoris* yeast expression system. Each dose contains 40 µg of HPV-16 L1 protein and 20 µg of HPV-18 L1 protein as the active ingredient. Walrinvax is administered as a two-dose regimen at 0 and 6 months for individuals 9 to 14 years of age and a three-dose regimen (0, 2, and 6 months) for individuals 15 years of age and older [20].

Walrinvax’s safety and immunogenicity have been established in Phase I and II clinical trials in women aged 9–45 [20]. A placebo-controlled Phase III study in China among 18–30 year olds demonstrated 78.6% (95% CI: 23.3–96.1) protection against HPV-16 and HPV-18-associated high-grade cervical lesions (CIN2+) after three doses [21]. Although indicating a significant effect on the results, the wide confidence interval raises, probably due to very early data collection and the limited number of cases observed. Longer follow-up periods and greater accumulation of cases are expected to provide a more definitive assessment of vaccine efficacy [20,21].

A double-blind, randomised, non-inferiority clinical trial published in 2023 evaluated the long-term immunogenicity of Walrinvax over a 36-month follow-up period in 900 participants. The study showed that antibody levels elicited by a two-dose regimen in adolescent girls (aged 9–14 years) were at least equivalent (non-inferior) to those elicited by a three-dose regimen in young adult women (aged 18–26 years) [15].

In 2022, the Chinese NMPA approved Walrinvax, and the WHO prequalified it in 2024. The vaccine was confirmed by the WHO prequalification as meeting global standards for quality, safety and efficacy, and is particularly important in its supply and distribution in Low and Middle Income Countries [14]. Despite its approval by the WHO and NMPA, Walrinvax has not yet received authorization from the FDA or the EMA. Although Walrinvax represents a cost-effective alternative, further clinical trials are needed to assess its long-term effectiveness in preventing precancerous lesions and HPV-associated cancers [7].

### 3.3. Cervavac (HPV-6/11/16/18, Quadrivalent Vaccine)

Cervavac^®^ is a quadrivalent human papillomavirus (HPV) vaccine developed by the Serum Institute of India Pvt. Ltd. (Pune, India) (SII). As the first indigenously developed HPV vaccine in India, it contains L1 VLPs of HPV types 6, 11, 16, and 18, produced using the *Hansenula polymorpha* expression system [8]. Cervavac^®^ was developed in collaboration with the Ministry of Biotechnology, the Biotechnology Industry Research Assistance Council, and the Bill & Melinda Gates Foundation. Cervavac^®^ is given as two doses at ages 9–14 years (0 and 6 months) and three doses at ages 15 years and above [22].

Cervavac was approved for commercial use by the Drug Controller General of India (DCGI) on 12 July 2022, following a randomized, multicenter Phase II/III clinical trial in which it demonstrated non-inferior immunogenicity compared to Gardasil [16]. Although direct clinical efficacy data on Cervavac’s effect in preventing HPV-associated diseases are not yet available, its comparable immunogenicity to Gardasil suggests that it may provide equivalent protection against genital warts (HPV-6/11) and precancerous cervical lesions (HPV-16/18). However, further long-term efficacy studies are needed to confirm these predictions.

## 4. Ongoing Trials

Despite the availability of bivalent (HPV16/18), quadrivalent (HPV6/11/16/18) and nonavalent (HPV6/11/16/18/31/33/45/52/58) vaccines, HPV vaccination coverage rates are not at the desired level even in Europe, and there are significant differences across countries. Although the nonavalent vaccine provides the broadest protection against HPV types responsible for approximately 90% of cervical cancers, it does not cover all high-risk types [23]. HPV vaccination has been integrated into numerous national immunisation programmes; however, global coverage remains suboptimal, with 31% of adolescent girls having received at least one dose as of 2024. Furthermore, completion rates are lower, with only 28% of girls worldwide receiving the last dose of the vaccine series [24]. This rate is well below the WHO’s target of 90% by 2030. Pricing and limited healthcare resources for HPV vaccines remain significant barriers to access, particularly in low-income countries. Advanced economic analyses suggest that HPV vaccination is cost-effective, particularly in areas with high cervical cancer incidence [25].

To lessen the burden of HPV-related cancer or preinvasive lesions, ongoing research focuses on next-generation vaccines. These new vaccine candidates aim to offer broader protection against oncogenic HPV types, thereby enhancing prevention strategies (13). In recent years, several biotechnology companies and research institutions, especially in China and India, have developed new HPV vaccines, including 4-valent, 9-valent, 11-valent, and 14-valent formulations. The current landscape of next-generation HPV vaccine candidates and their clinical trial phases are summarized in Table 2.

The quadrivalent HPV vaccine candidate developed by Chongqing Bovax Biotechnology Co., Ltd., Chongqing, China, which has completed Phase I safety and immunogenicity studies, contains L1 VLPs derived from HPV types 6, 11, 16, and 18. The same candidate, developed by Chongqing Bovax, demonstrates the inclusion of these proteins and has shown promising results. Bovax’s quadrivalent vaccine demonstrated non-inferior immunogenicity to Gardasil in a randomised, blinded Phase III trial involving 1680 women aged 20 to 45 years. The vaccine does not yet have regulatory approval, and direct clinical efficacy data, including information on preventing infection and lesions, are not yet available [26].

The nonavalent HPV vaccine developed by Shanghai Bovax Biotechnology Co., Ltd., Shanghai, China has similar content to Gardasil-9 (13). In Phase III immunogenicity studies, the vaccine produced by Shanghai Bovax was shown to have a non-inferior immune response to Gardasil. (36) An additional placebo-controlled Phase III study assessing the vaccine’s efficacy in 8000 women (NCT04422366) is currently ongoing. Two more studies are underway: an immune bridging study involving 1200 participants aged 9–19 years (NCT04895020) and a separate safety and dosing study in males aged 9–45 years (13). Shanghai Bovax’s 9-valent HPV vaccine has not yet received regulatory approval.

Xiamen Innovax Biotech Co., Ltd., Xiamen, China (in collaboration with Beijing Wantai Biological Pharmacy Enterprise Co., Ltd., Beijing, China) 9-valent HPV vaccine candidate targets HPV types 6, 11, 16, 18, 31, 33, 45, 52 and 58. Innovax has previously developed an approved *E. coli*-based bivalent HPV vaccine, Cecolin^®^, and they are also producing *E. coli*-based [21]. The Innovax 9-valent vaccine was initially evaluated in a Phase I trial, demonstrating good tolerability and a strong immune response [27]. A randomized, double-blind, head-to-head Phase III study was conducted in women aged 18 to 49 years. The study, conducted in 2023, directly compared its immunogenicity to Gardasil9 and confirmed noninferiority in type-specific antibody responses [28]. To assess long-term efficacy and safety, a large, placebo-controlled Phase III trial (NCT04537156) is ongoing in 9237 women aged 18–45 years. In addition, the immune response of the vaccine in adolescents (9–17 years) compared to young adults is being evaluated in an immune-bridging Phase III study (NCT05056402) [21]. Innovax’s 9-valent HPV vaccine (Cecolin 9) was licensed by the NMPA in 2025, becoming China’s first locally developed and approved 9-valent vaccine. Given Cecolin^®^’s previous approval by the WHO, a similar process is expected for Cecolin^®^9.

GlaxoSmithKline Biologicals, Rixensart, Belgium (GSK) is developing a nonavalent HPV vaccine candidate targeting HPV types 6, 11, 16, 18, 31, 33, 45, 52 and 58. The vaccine is still being tested on humans. This is happening in two phases. In the first phase, the drug is being tested on women aged 16 to 26. The testing checks how safe the drug is, how the body responds to it and what dosage is needed. It has not been approved, and Phase III trials and regulatory submissions are expected to be entered in the later stages of development [29].

The 9-valent HPV vaccine candidate developed by Beijing Health Guard is produced with similar content to Cecolin9 and Gardasil 9-valent vaccines, and L1 VLPs are based on *E. coli* [21]. A 2023 study demonstrated the stability of the vaccine, showing that it remained intact for up to 72 months at 2–8 °C and at least 12 months at 25 °C. These findings can be considered as a logistical advantage in transporting the vaccine from production sites to low-income countries [30]. As of now, there are two parallel phase III studies ongoing in China evaluating the 9-valent HPV vaccine produced by Beijing Health Guard Biotechnology Co., Ltd., Beijing, China. The first study is a placebo-controlled study on efficacy, safety, and immunogenicity in approximately 12,000 healthy women (NCT05668572). Therefore, since the vaccine is still in Phase III development (NCT05662020), there is no clinical data to evaluate yet. The second study is an immune bridging study to evaluate immune responses and safety in 2750 participants aged 9–26. The results of the studies for this vaccine, which is a next-generation nonavalent vaccine, are awaited [21].

Walvax Biotechnology Co., Ltd., Kunming, China, the company responsible for manufacturing Walrinvax™, is currently developing a 9-valent HPV vaccine candidate to provide broader protection [21]. In preclinical studies in experimental animals, Walrinwax’s 9-valent vaccine demonstrated immunogenicity similar to Gardasil9 and higher levels of neutralising antibodies against certain HPV types [31]. The investigational vaccine is currently being tested for safety and immunogenicity in a Phase III trial (NCT05580341) involving 1200 participants aged 16–26 years. The vaccine remains in early Phase III development as clinical efficacy data are not yet available.

Jiangsu Recbio Technology Co., Ltd., Taizhou, China is developing a 9-valent HPV vaccine known as REC603, designed to target HPV types 6, 11, 16, 18, 31, 33, 45, 52, and 58. The vaccine is currently being evaluated in one of the large HPV vaccine trials in China (CTR20210947). A placebo-controlled, multicenter Phase III trial involving 16,050 healthy women is ongoing, and results from this study are awaited [21].

Republic of Korea-based SK Bioscience Co., Ltd., Seongnam, Republic of Korea is developing a quadrivalent HPV vaccine targeting HPV types 6, 11, 16 and 18. The vaccine is currently undergoing a Phase I/II clinical trial in Republic of Korea for safety and dose response in healthy females aged 9–26 years. The same company is conducting research on a 10-valent HPV vaccine after preclinical studies showed that an ssRNA-based adjuvant boosted immunity [32]. The quadrivalent vaccine produced by SK Bioscience is still in clinical development and has not yet received regulatory approval. Research and development teams are working on a 10-valent candidate with a target completion date of 2027 [29].

The 11-valent HPV vaccine, developed by the National Vaccine and Serum Institute of China (NVSI), aims to provide broader protection by containing two high-risk HPV types, 59 and 68, in addition to the 9-valent vaccines [33]. The NVSI 11-valent HPV vaccine entered clinical development in 2022 and is currently undergoing a randomised Phase III study (NCT05262010) in China with approximately 13,500 participants aged 9–45 years. Results of the study are expected in 2026.

Sinocelltech Group Ltd., Beijing, China is collaborating with China NVSI to develop the world’s first 14-valent HPV vaccine (SCT1000). This candidate vaccine is in addition to Gardasil9 by including five additional high-risk HPV types (35, 39, 51, 56, 59). Preclinical studies in mouse models have shown strong immunogenicity and stability, with robust neutralising antibody responses against all 14 target HPV types [34]. As of 2023, SCT1000 has advanced to Phase III (NCT06041061) to evaluate efficacy, safety, and immunogenicity in an ongoing study in China involving 18,000 healthy women aged 18–45 [33]. In addition, Shengda Bio is currently developing a 15-valent HPV vaccine that targets HPV-68 in addition to the 14 HPV types covered by SCT1000 [33].

## 5. L2 Vaccines-Future of Vaccine Technology

The L2 protein is a minor but functionally important component of the HPV capsid. Unlike L1, L2 does not self-assemble into virus-like particles and is only transiently exposed during viral entry [35,36]. L2 proteins are going to be broadly protective against different HPV types [36,37,38,39,40]. Studies on the N-terminal region of L2 have shown that it can induce broadly cross-protection (but low-titer antibodies) against various mucosal and cutaneous HPV types, which is not observed with L1-based vaccines [35,38,39]. The induced immunity varies in its effectiveness against different HPV types depending on the level of antibodies generated [41].

The L2 minor capsid protein plays an important role in facilitating efficient viral entry into host cells [42]. Although L2-based vaccines are primarily being developed as prophylactic strategies, the biological properties of L2 have also prompted exploratory research into its use as a component of experimental vaccine platforms. In particular, L2 has been incorporated into fusion constructs together with early HPV proteins, such as E6 and E7, to enhance antigen presentation and immune recognition. For example, vaccine candidates such as TA-CIN and TA-GW have demonstrated HPV-16-specific T-cell responses directed against L2E6E7 fusion antigens [43]. However, clinical studies have not consistently shown a direct correlation between immunological responses and clinical efficacy, highlighting the need for further investigation [44].

Current HPV vaccines require specific storage conditions. For example, Cervarix™ and Gardasil-9 are recommended to be stored at temperatures between 2 °C and 8 °C [39,42]. Some of the L2-based vaccines under development are known to be produced in powder form, which allows for long-term storage at room temperature [45,46,47]. This characteristic could facilitate vaccination programs in countries with inadequate storage facilities [39]. Additionally, the potential for lower production costs compared to L1-based vaccines represents another advantage of L2-based vaccines [39].

Nevertheless, several challenges remain for L2-based vaccines. The strong immune response generated against L1 VLPs is not observed for L2 peptides [38,47], raising concerns about the strength and duration of immunity conferred by L2-based vaccines [48]. For example, the RG1-VLP vaccine, which incorporates the RG1 epitope—a highly conserved N-terminal region of the HPV L2 minor capsid protein—has demonstrated protection against multiple HPV types for at least one year [40]. Various strategies, such as the use of adjuvants, fusion with immunostimulatory agents, presentation by HPV L1-VLP, and concatemeric peptides, are being explored to achieve durable and adequate immunity with L2-based vaccines [36,39].

Taken together, L2-based HPV vaccines represent an important advance in next-generation prophylactic strategies; however, they are inherently limited to infection prevention and are not intended to address established HPV-associated disease. To complement prophylactic vaccination, a distinct and rapidly evolving field has therefore emerged focusing on therapeutic HPV vaccines specifically designed for disease control and treatment.

Therapeutic HPV vaccines are immune-based interventions that aim to eliminate established HPV infections and HPV-driven premalignant or malignant diseases by inducing antigen-specific cellular immunity against the viral oncoproteins E6 and E7, which are selectively expressed in transformed cells [42]. Unlike prophylactic HPV vaccines, which prevent infection by inducing neutralising antibodies against capsid proteins, therapeutic vaccines target HPV-infected or transformed cells by activating HPV-specific CD8^+^ cytotoxic T cells and CD4^+^ helper T cells. Accordingly, therapeutic HPV vaccines are primarily positioned within secondary prevention for the treatment of high-grade cervical intraepithelial neoplasia (CIN), and potentially within tertiary prevention when used as adjuncts in established HPV-related malignancies.

Although there is currently no approved therapeutic HPV vaccine for routine clinical use, multiple vaccine platforms have demonstrated encouraging efficacy and immunogenicity in phase I–III clinical trials [42]. These platforms include DNA-based vaccines (such as VGX-3100 and GX-188E), peptide-based vaccines (ISA101), and viral and bacterial vector-based approaches [49,50]. To date, therapeutic HPV vaccines have demonstrated the greatest clinical efficacy in the treatment of high-grade cervical intraepithelial neoplasia (CIN2/3). Several vaccine candidates have been shown to induce histological regression and viral clearance without the need for surgical excision. However, their effectiveness as a monotherapy in the treatment of advanced or invasive cancers remains limited [33]. Nevertheless, combination strategies incorporating immune checkpoint inhibitors, chemotherapy or radiotherapy appear to enhance anti-tumour efficacy. Looking ahead, therapeutic HPV vaccines offer key advantages, including organ preservation, reduced surgical morbidity and durable, disease-specific immune memory, and they are expected to be used primarily within personalised combination immunotherapy frameworks rather than as standalone agents [33,42]. DNA-based vaccines such as VGX-3100 and GX-188E have demonstrated histological regression and viral clearance in phase II–III trials involving patients with CIN2/3. Peptide-based vaccines, including ISA101, have shown the ability to induce robust HPV-specific T-cell responses and have been evaluated both as monotherapy and in combination with immune checkpoint inhibitors in phase I–II studies. In addition, viral vector–based approaches (e.g., TG4001, ADXS11-001) have reached early- to mid-phase clinical testing with encouraging immunogenicity and acceptable safety profiles [33].

## 6. Cost-Effectiveness of HPV Vaccines

Previous research has shown that HPV vaccines are cost-effective [51]. HPV vaccination has been proven to be cost-effective in high-income countries [51]. Furthermore, studies in low- and middle-income countries indicate that vaccination is cost-effective due to the GAVI vaccination support programme [52]. A study in the low-income Philippines found that the 2-valent and 4-valent vaccines were cost-effective, whereas the 9-valent vaccine was not [53].

A cost-effectiveness study was also conducted on the more recent Cecolin vaccine. This study considered the average HPV infection rate in the population in China, the incidence of CIN and above lesions, their progression, the incidence of cervical cancer, and the associated treatment costs. In the comparison group, the cost of vaccinating girls aged 9–14 years with 2 doses of the vaccine was evaluated along with the HPV screening test. The Cecolin vaccine was found to be more cost-effective than the previously approved Cervarix vaccine because it was about half the cost and had similar efficacy [54,55].

In addition to Cecolin, the quadrivalent vaccine Cervavac and the bivalent vaccine Walrinvax have not yet been evaluated in formal cost-effectiveness analyses. Nevertheless, these vaccines are expected to improve the cost-effectiveness of human papillomavirus vaccination programmes, mainly because of their lower anticipated production and procurement costs and their potential to enhance market competition and vaccine supply.

## Figures and Tables

**Table 1:** HPV vaccines currently approved.

	Bivalent			Quadrivalent		Nonavalent	
	Cervarix	Cecolin	Walrinvax	Gardasil	Cervavac	Gardasil 9	Cecolin 9
**Manufacturer**	GlaxoSmithKline Biologicals, Rixensart, Belgium	Xiamen Innovax Biotech Co., Ltd., Xiamen, China	Yuxi Zerun Biotechnology Co., Ltd., Yuxi, China	Merck & Co., Inc., Kenilworth, NJ, USA	Serum Institute of India Pvt. Ltd., Pune, India	Merck & Co., Inc., Kenilworth, NJ, USA	Xiamen Innovax Biotech Co., Ltd., Xiamen, China
**Year of approval**	2008	2021	2022	2006	2022	2014	2025
**Approving authority**	FDA, USA	NMPA-China	NMPA-China	FDA, USA	DCGI, India	FDA, USA	NMPA (China)
**HPV types covered**	HPV 16 and 18	HPV 16 and 18	HPV 16 and 18	HPV 6, 11, 16, and 18	HPV 6, 11, 16, and 18	HPV 6,11,16,18,31,33, 45,52,and 58	HPV 6,11,16,18,31,33, 45,52,and 58
**Recombinant expression system**	Insect Cell-Baculovirus	*Escherichia coli*	*Pichia pastoris*	*Saccharomyces cerevisiae* (yeast)	*Hansenula polymorpha*	*Saccharomyces cerevisiae* (yeast)	*Escherichia coli*
**Risk reduction of**	Cervical cancer, CIN2/3, AIS, CIN1 caused by HPV-16/18	Cervical cancer, CIN2/3, AIS, CIN1 caused by HPV-16/18	Cervical cancer, CIN2/3, AIS, CIN1 caused by HPV-16/18	Cervical, vulvar, vaginal, anal cancer, Genital warts (condyloma acuminata) and precancerous or dysplastic lesions	Cervical, vulvar, vaginal, anal cancer, Genital warts (condyloma acuminata) and precancerous or dysplastic lesions	Cervical, vulvar, vaginal, oropharyngeal, anal cancer, precancerous or dysplastic lesions and Genital warts (condyloma acuminata)	Cervical cancer, CIN2/3, AIS, and HPV-related precancerous lesions
**L1 Virus-Like Particle (VLP) Content**	20/20 μg	40/20 μg	40/20 μg	20/40/40/20 μg	20/40/40/20 μg	30/40/60/40/20/20/20/ 20/20 μg	30/40/60/40/20/20/20/ 20/20 μg
**Adjuvant**	500 μg aluminum hydroxide, 50 μg 3-Odeacylated- 4′- monophosphoryl lipid A	Aluminium hydroxide	225 μg aluminum phosphate	225 μg aluminum hydroxyphosphate sulfate	500 µg Aluminium hydroxide	500 μg amorphous aluminum hydroxyphosphate sulfate	Aluminium hydroxide
**Targeted age range (years)**	9–25	9–45	9–30	9–45	9–26	9–45	9–45
**Vaccination regimen**	9–14 years old: 2 doses (0 and 6 months) 15–45 years old: 3 doses (0, 1 and 6 months)	9–14 years old: 2 doses (0 and 6 months) 15–45 years old: 3 doses (0, 1 and 6 months)	9–14 years old: 2 doses (0 and 6 months) 15–30 years old: 3 doses (0, 2 and 6 months)	9–14 years old: 2 doses (0 and 6~12 months) * 15–45 years old: 3 doses (0, 2 and 6 months) **	9–14 years old: 2 doses (0 and 6 months) 15–26 years old: 3 doses (0, 2 and 6 months)	9–14 years old: 2 doses (0 and 6~12 months) * 15–45 years old: 3 doses (0, 2 and 6 months) **	9–17 years old: 2 doses (0 and 6 months) 18–45 years old: 3 doses (0, 1–2 and 6 months)

* In a two-dose schedule of HPV vaccine, the minimum interval is 5 months between the first and second dose. third dose. ** In a three-dose schedule of HPV vaccine, the minimum intervals are 4 weeks between the first and second dose, 12 weeks between the second and third dose.

**Table 2:** Ongoing Clinical Trials of Next-Generation Prophylactic HPV Vaccines.

Manufacturer	Valency	Sample Size (n)	Trial Phase	Population (Age)	Design	Primary Outcomes	Status/Registry
**Chongqing Bovax Biotechnology Co., Ltd., Chongqing, China**	4-valent	1680	Phase III	Women aged 20–45 years	Randomized, blinded	Immunogenicity, safety	Phase III completed
**Shanghai Bovax Biotechnology Co., Ltd., Shanghai, China**	9-valent	~8000	Phase III	Women aged 20–45 years	Placebo-controlled	Efficacy, safety	NCT04422366
**Shanghai Bovax Biotechnology Co., Ltd., Shanghai, China**	9-valent	1200	Phase III	Adolescent women (9–19 years) and women aged 20–45 years	Immunobridging	Immunogenicity	NCT04895020
**Xiamen Innovax Biotech Co., Ltd., Xiamen, China**	9-valent	487	Phase III	Women aged 18–49 years	Head-to-head vs Gardasil9	Immunogenicity	Completed
**Xiamen Innovax Biotech Co., Ltd., Xiamen, China**	9-valent	9237	Phase III	Women aged 18–45 years	Placebo-controlled	Efficacy, safety	NCT04537156
**Xiamen Innovax Biotech Co., Ltd., Xiamen, China**	9-valent	—	Phase III	Adolescent women aged 9–17 years	Immunobridging	Immunogenicity	NCT05056402
**GlaxoSmithKline Biologicals, Rixensart, Belgium**	9-valent	—	Phase I–II	Women aged 16–26 years	Dose-escalation, randomized	Safety, immunogenicity, dose-finding	Ongoing
**Beijing Health Guard Biotechnology Co., Ltd., Beijing, China**	9-valent	~12,000	Phase III	Women aged 20–45 years	Placebo-controlled	Efficacy, safety	NCT05668572
**Beijing Health Guard Biotechnology Co., Ltd., Beijing, China**	9-valent	2750	Phase III	Women aged 9–26 years	Immunobridging	Immunogenicity	NCT05662020
**Walvax Biotechnology Co., Ltd., Kunming, China**	9-valent	1200	Phase III	Women aged 16–26 years	Randomized	Safety, immunogenicity	NCT05580341
**Jiangsu Recbio Technology Co., Ltd., Taizhou, China**	9-valent	16,050	Phase III	-	Multicenter, placebo-controlled	Efficacy	CTR20210947
**SK Bioscience Co., Ltd., Seongnam, Republic of Korea**	4-valent	Not disclosed	Phase I/II	Women aged 9–26 years	Dose-escalation	Safety, immunogenicity	Ongoing
**National Vaccine and Serum Institute (NVSI), Beijing, China**	11-valent	~13,500	Phase III	Women aged 9–45 years	Randomized	Efficacy, safety	NCT05262010
**Sinocelltech Group Ltd., Beijing, China**	14-valent	~18,000	Phase III	Women aged 18–45 years	Placebo-controlled	Efficacy, safety	NCT06041061

## Data Availability

Data are contained within the article.
